# Simple Smartphone-Based Guiding System for Visually Impaired People

**DOI:** 10.3390/s17061371

**Published:** 2017-06-13

**Authors:** Bor-Shing Lin, Cheng-Che Lee, Pei-Ying Chiang

**Affiliations:** 1 Department of Computer Science and Information Engineering, National Taipei University, New Taipei City 23741, Taiwan; bslin@mail.ntpu.edu.tw (B.-S.L.); a6214123@gmail.com (C.-C.L.); 2 Department of Computer Science and Information Engineering, National Taipei University of Technology, Taipei 10608, Taiwan

**Keywords:** smartphone-based, guiding system, visually impaired, deep learning, obstacle recognition, mobile device application

## Abstract

Visually impaired people are often unaware of dangers in front of them, even in familiar environments. Furthermore, in unfamiliar environments, such people require guidance to reduce the risk of colliding with obstacles. This study proposes a simple smartphone-based guiding system for solving the navigation problems for visually impaired people and achieving obstacle avoidance to enable visually impaired people to travel smoothly from a beginning point to a destination with greater awareness of their surroundings. In this study, a computer image recognition system and smartphone application were integrated to form a simple assisted guiding system. Two operating modes, online mode and offline mode, can be chosen depending on network availability. When the system begins to operate, the smartphone captures the scene in front of the user and sends the captured images to the backend server to be processed. The backend server uses the faster region convolutional neural network algorithm or the you only look once algorithm to recognize multiple obstacles in every image, and it subsequently sends the results back to the smartphone. The results of obstacle recognition in this study reached 60%, which is sufficient for assisting visually impaired people in realizing the types and locations of obstacles around them.

## 1. Introduction

According to a 2012 statistical report from the World Health Organization [1], approximately 285 million people worldwide are blind or have amblyopia, 246 million of whom have serious vision problems. People with amblyopia have less viewable areas than the normal population. Some people with amblyopia have distorted vision or can only sense light and shadows, without being able to discern objects in front of them. Moreover, blind people depend on their experiences or other senses to walk.

Visually impaired people usually have problems walking and avoiding obstacles in their daily lives. Traditionally, such people use guide canes to detect obstacles in front of them. Thus, visually impaired people cannot exactly know what types of obstacles are in front of them and must only depend on guide canes and experiences to walk safely and in the desired path. Furthermore, when in unfamiliar environments, visually impaired people often require assistance in the form of volunteers to guide them through the surrounding environment. Visually impaired people cannot entirely depend on a guide cane to become familiar with their surroundings or react quickly to unforeseen circumstances.

When encountering obstacles, visually impaired people must only rely on their experiences to react because they may not know what the obstacles are. However, some obstacles on the road cannot be predicted, such as a parked bicycle or a resting dog. For visually impaired people, reacting quickly to avoid such obstacles by relying solely on experiences is difficult. Therefore, such obstacles could engender danger for an unaccompanied visually impaired person. 

Many studies have investigated navigation for blind people. According to these studies, devices and recognition methods can be divided into the following three categories: electronic travel aids (ETAs), electronic orientation aids (EOAs), and position locator devices (PLDs), which all have advantages and disadvantages. These categories are described as follows:

ETAs are general assistant devices to help visually impaired people avoid obstacles [2,3,4,5,6,7]. The sensing inputs of ETAs are mainly classified into depth camera, general camera, radio frequency identification (RFID), ultrasonic sensor, and infrared sensor. Regarding depth camera recognition systems, Microsoft Kinect is usually used as the main recognition hardware in such systems [2,3,4,5,6]. Kinect captures images of the user’s field of vision and analyzes the depth data of the images to predict the presence of obstacles ahead and alert the user of any such obstacles. Compared with purely two-dimensional images, depth images can provide more information about obstacles. However, Kinect cannot be used in environments with strong light. Moreover, it can determine only the presence of obstacles ahead [5], or recognize a few types of obstacles in few related studies [6]. Furthermore, depth cameras are more expensive than standard cameras and excessively large for convenient use; therefore, depth camera recognition systems are seldom used in actual applications. Regarding general camera recognition systems [7], such systems are designed to recognize tactile or obstacle images. General cameras do not to provide the depth information so that such systems cannot determine the distance from user to obstacle. General images usually are processed to detect only the obstacles ahead or guide visually impaired people walking along the tactile marks. General images similar to depth images need complex computing to hardly be implemented as a wearable guiding device or a guide cane.

Detecting distance sensors, such as RFIDs, ultrasonic sensors and infrared sensors, are usually used to modify guide canes [8,9,10,11,12,13,14,15,16,17,18,19,20]. Regarding the RFID sensing method, a RFID reader module is installed on a guide cane, and a large quantity of RFID tags are positioned underground for positioning and navigation. Such assistive devices are not widely used and are difficult to apply in real-life situations; in addition, without large quantities of RFID chips being positioned underground in advance, the system cannot work. Regarding ultrasonic [14,15,16,17] and infrared [18,19,20] sensors, they are cheap and easily implemented into a guide cane. These kinds of sensors can precisely determine the distance and notify the visually impaired people there are obstacles in front, but they cannot recognize the obstacle categories. In more advanced design, multiple infrared sensors [19] can detect special cases such as stairs, but still cannot detect the more complex shape of the obstacle.

EOAs are designed to aid visually impaired people for finding their way in an unknown environment. EOA systems usually need much environmental information to analyze the scope of unknown environment. A combination of a camera and other multiple sensors is usually used to get more information to draw the shapes of passageway and obstacles [5,6]. Thus these systems may provide a guiding service and a recognition result of a few types of obstacles. The drawback of EOAs is that they need more complex computing to hardly be realized as a real-time and lightweight guiding device.

PLDs are used to determine the precise position of its holder such as devices that use global positioning system (GPS) and geographic information system (GIS) technologies [15,17,20,21,22]. GPS and GIS-based guiding systems for blind people with user input interfacing (such as voice) intellectually find the current location and give the alert to the blind people if he arrives at his destination area. A pure GPS and GIS-based navigation systems for general people are easily used to guide user from current location to destination. However, the pure GPS and GIS-based navigation systems do not completely work in visually impaired people because the system cannot help user to avoid the obstacles in front of them. Thus these kinds of system usually need to collocate with other sensors to detect the obstacles. These systems need receive the signals from GPS satellites, so that they only can be used in outdoor, but not in indoor.

In summary, according to the implementations in previous studies, assistive devices for navigation for visually impaired people still focus on location and distance sensing, but cannot warn users about the types of obstacles in front of them. Moreover, distance sensing cannot provide additional information to help visually impaired people to understand their surroundings. Therefore, the practicability of such assistive devices is very low. Some solutions using RFID chips are expensive and vulnerable to damage from the sun and rain. Therefore, the current study proposes a navigation system for visually impaired people; this system employs a smartphone and deep learning algorithms to recognize various obstacles. The proposed system is not limited to specific indoor or outdoor environments and does not require the positioning of RFID chips in advance. Thus, the proposed system not only increases the number of available locations, but also provides more information for visually impaired people about their surroundings.

## 2. Methodology 

The proposed navigation system employs a smartphone to continually capture images of the environment in front of a user and perform image processing and object identification to inform the user of the image results. According to these results, the user can gain a more comprehensive understanding of the surroundings. This system enables visually impaired people to not only know the rough direction and distance to an obstacle, but also know what the obstacle is. 

A university scene was used as an example to illustrate our proposed system. When a visually impaired user is walking on a campus, two scenarios may occur. In the first scenario, the visually impaired user comes to the campus for the first time. Hence, the user is not familiar with the university’s environment and thus walks at a reduced speed. In this scenario, the user requires high recognition accuracy. Accordingly, the system mechanism called “stable mode” is provided for achieving high recognition accuracy. Under the stable mode, the user can become familiar with the environment quickly by obtaining precise notifications with higher recognition rates than the other mode. In the second scenario, the visually impaired user is walking through a crowded area or an area with many obstacles such as bicycles parked randomly in front of the university building or dogs resting in the user’s path. The user has a high probability of colliding with some of the obstacles. In this scenario, the system requires a high recognition speed to alert the user about the environment in front of him or her. Accordingly, the system mechanism called “fast mode” is provided. This mode provides the user with a high quantity of obstacle alerts within a short period. The processing time of the fast mode is shorter than that of the stable mode. According to the preceding scenarios, the proposed system provides stable and fast modes for corresponding circumstances. Through one of these two modes, users can obtain information with varying degrees of accuracy and recognition speed for individual walking situations. 

### 2.1. System Architecture

**Figure 1:**
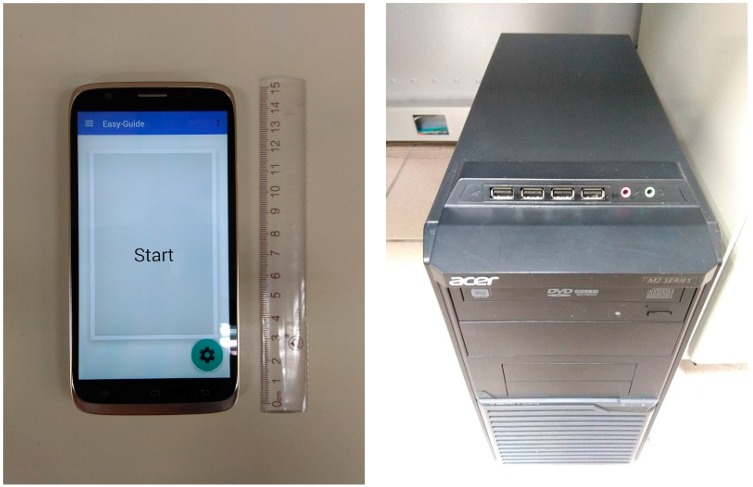
Photograph of the smartphone (**left**) and server (**right**).

The proposed system consists of smartphone and server side. The smartphone side consists of a smartphone equipped with a back camera and a network module. This study used the Infocus M320 smartphone (M320, Infocus, Tigard, OR, USA) for development and testing. For the specification of the smartphone, the width and length of the smartphone are 77.4 mm and 150.5 mm respectively. The weight of the smartphone is 191 g. For the battery aspect, the capacity is 3100 mAh. On the other hand, the specification of server hardware is a personal computer (Veriton M2611G, Acer Inc., New Taipei City, Taiwan) which is equipped with central processing unit (CPU) Intel Core i7-3370 3.4 GHz, 8 GB DRAM, and a graphics processing unit (GPU) (GT640-2GD3, ASUS Inc., Taipei, Taiwan) that can run Compute Unified Device Architecture (CUDA) to execute deep computing. Figure 1 shows the photograph of the smartphone and server. 

**Figure 2:**
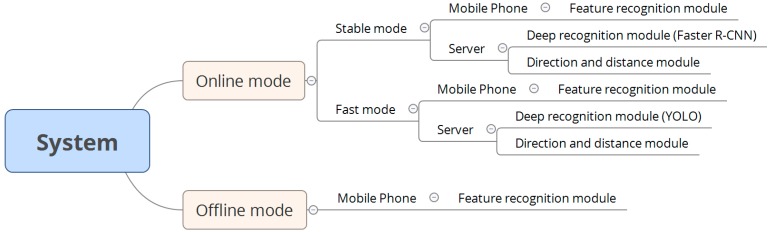
Relationships between various modules and modes.

The relationships between various modules and modes in the system are shown in Figure 2. Our system has online and offline modes. The online mode contains an option to switch between the stable mode and fast mode. In an environment with a stable Internet connection, the system automatically executes the stable mode (the default mode) to maintain system availability. In the stable mode, the smartphone contains a feature recognition module, and the server contains a deep recognition module, which is based on the faster region convolutional neural network (Faster R-CNN) algorithm, and a direction and distance module. Furthermore, in the fast mode, the smartphone contains a feature recognition module, and the server contains a deep recognition module, which is based on the you only look once (YOLO) algorithm, and a direction and distance module. The feature recognition modules in the smartphone are identical in the online and offline modes, and the direction and distance modules installed on the server are identical in the stable and fast modes. By contrast, the two deep recognition modules in the different modes are not the same: The Faster R-CNN algorithm is executed in the stable mode, whereas the YOLO algorithm is executed in the fast mode.

**Figure 3:**
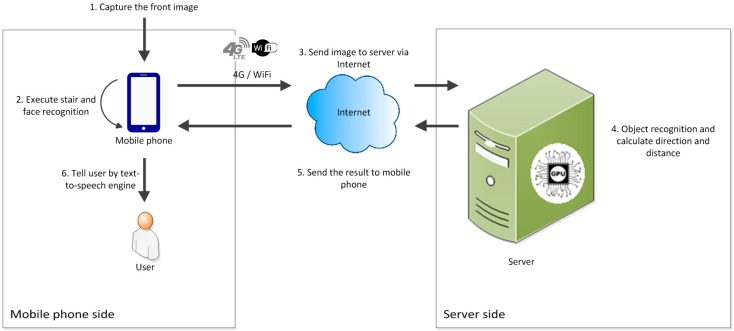
Data and notification message transmission diagram in the online mode.

Figure 3 shows the working processes of the online mode. At the beginning of the process, the smartphone captures images of the scene in front of the user continuously. After the images are generated, they are sent to the feature recognition module on the smartphone for “face and stair recognition” to be executed. Meanwhile, the images are also sent to the backend server via the Internet. After the image being sent to the server, the image storage will be cleared. The flushing memory design can guarantee the personal confidentiality even if smartphone is lost. After the images are received by the server, they are sent to the deep recognition module and direction and distance module for the results to be obtained. Subsequently, the server sends the results including the obstacle type, direction, and distance from the user back to the smartphone. Then the memory of server which stores the recognition image will be cleaned. Finally, the smartphone integrates the results and informs the user through voice notifications.

**Figure 4:**
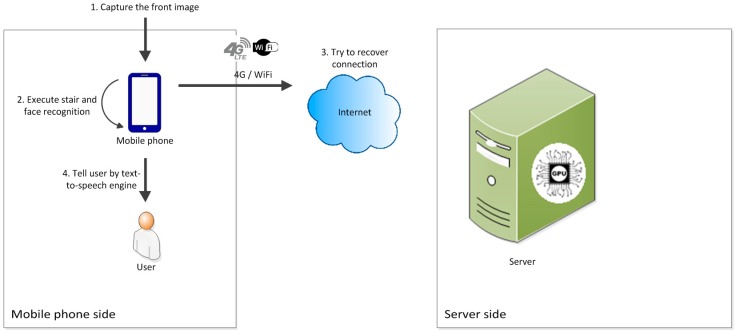
Diagram of data and notification message transmission in the offline mode.

When a user passes through an underpass or area with poor signal coverage, the smartphone’s connection program automatically disconnects and switches to offline mode. In this mode, the smartphone still captures images of the scene in front of the user continuously and sends them to the feature recognition module on the smartphone. After recognition, the smartphone directly provides the results to the user. The smartphone simultaneously continues attempting to connect to the backend server. The smartphone switches to online mode after successfully connecting to the server. Figure 4 shows the offline mode working processes.

### 2.2. Software Processes

This section first describes the port negotiation mechanism and subsequently introduces the processes on the smartphone and server sides.

#### 2.2.1. Port Negotiation Mechanism

When the user operates the system, the Internet connection might be interrupted because of a weak signal. Two standard approaches can be employed to deal with this problem. One of these approaches involves the hand-shaking method. In this method, the smartphone and server allocate extra resources to verify whether the connection has been interrupted. In the second approach, the server sets a reasonable termination time after a connection is established. If the server does not receive any data during this period, the connection is considered to be interrupted. Upon disconnection, the smartphone switches to offline mode and attempts to switch back to online mode after the connection has been recovered.

**Figure 5:**
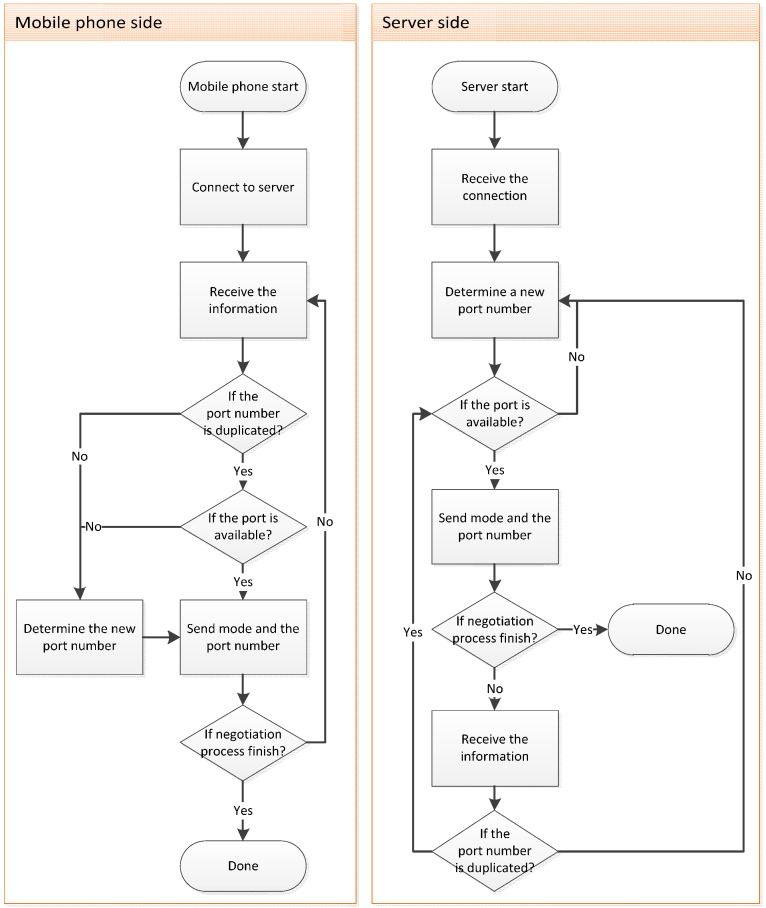
Smartphone- and server-based port negotiation processes.

Smartphone resources are limited. If the system allocates extra resources to perform periodical hand shaking, its efficiency deteriorates. Consequently, we adopted the second approach for the proposed system. When the smartphone attempts to establish a connection, both the smartphone and the server negotiate the connecting port number through the port negotiation mechanism. Subsequently, the smartphone and server close the connection after the negotiation has been completed, and the smartphone establishes the connection request toward the corresponding port number. When an unusual interruption occurs, the allocated port does not unregister and cannot be immediately recycled by the system. Consequently, the smartphone and server negotiate a new port number while the connection is still interrupted. Through this mechanism, the smartphone can avoid connecting to the same connection port and being refused by the server. In addition, the system periodically recycles the ports that are registered but have been idle for a certain period. The port negotiation procedure is shown in Figure 5.

#### 2.2.2. Process on the Smartphone Side

**Figure 6:**
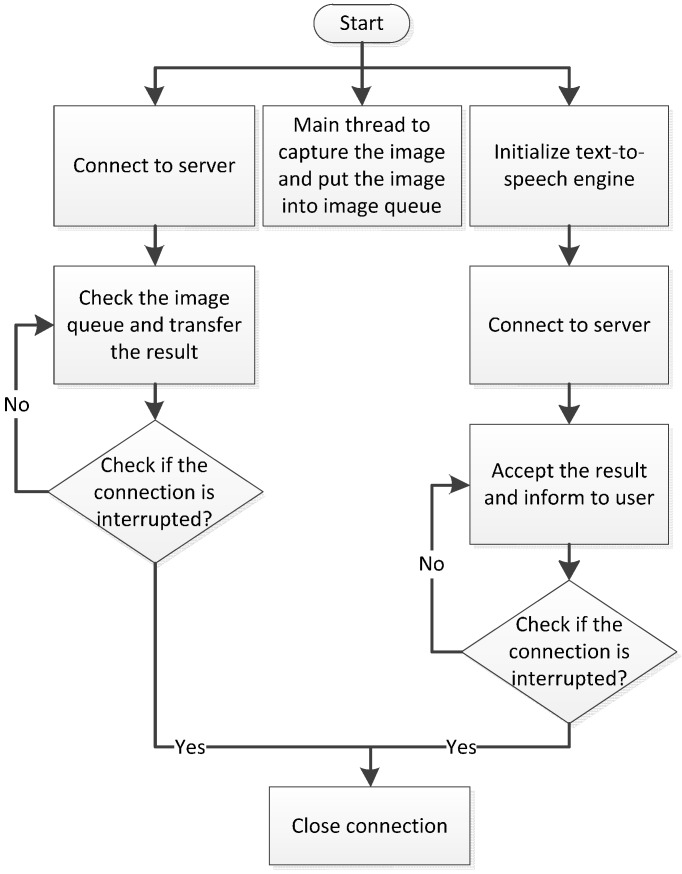
TCP transmission.

The operating process of software on the smartphone side is shown in the Figure 6. First, the port negotiation mechanism is implemented when the user starts using the device. Subsequently the software adopts a corresponding transmission protocol according to the user’s settings. In the proposed system, the Transmission Control Protocol (TCP) and Real-time Transport Protocol (RTP) are used to transfer the information in stable and fast modes, respectively. The RTP is the standard protocol for transmitting a large quantity of images, and it is the applied protocol based on the user datagram protocol (UDP). Because the UDP does not guarantee the order or correctness of transmissions, the RTP is more effective for overcoming the disadvantages of the UDP. Through the time-stamp design of the RTP, the server can deal with the corresponding image in an orderly fashion.

**Figure 7:**
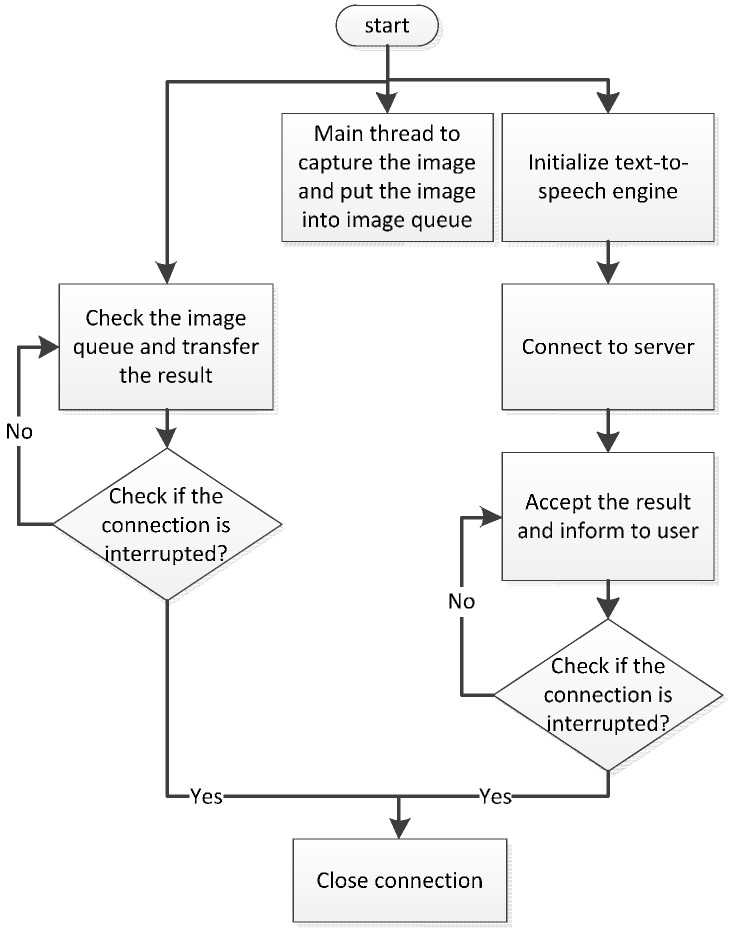
RTP transmission.

In the stable mode, the smartphone starts to establish an image transmission request. After a connection has been built, the smartphone starts to transmit the image to the server. Meanwhile, the smartphone initializes the Text-to-Speech (TTS) engine and establishes a request to receive results. If this connection is successful, the smartphone continues to receive the recognition results passed from the server. Concurrently, image capturing continues to gain the continuous input of the process. The entire procedure of the stable mode is shown in Figure 6. The only difference between the stable mode and fast mode is that the smartphone does not make image transmission requests in the fast mode. Under such circumstances, the smartphone directly transmits the RTP package to the server. Concurrently, the smartphone initializes the TTS engine and launches the request to receive results. When the smartphone’s connection is interrupted, the image recognition process is terminated. The process of the fast mode is shown in Figure 7. After both connections have been interrupted, the smartphone performs port negotiation again.

#### 2.2.3. Process on the Server Side

**Figure 8:**
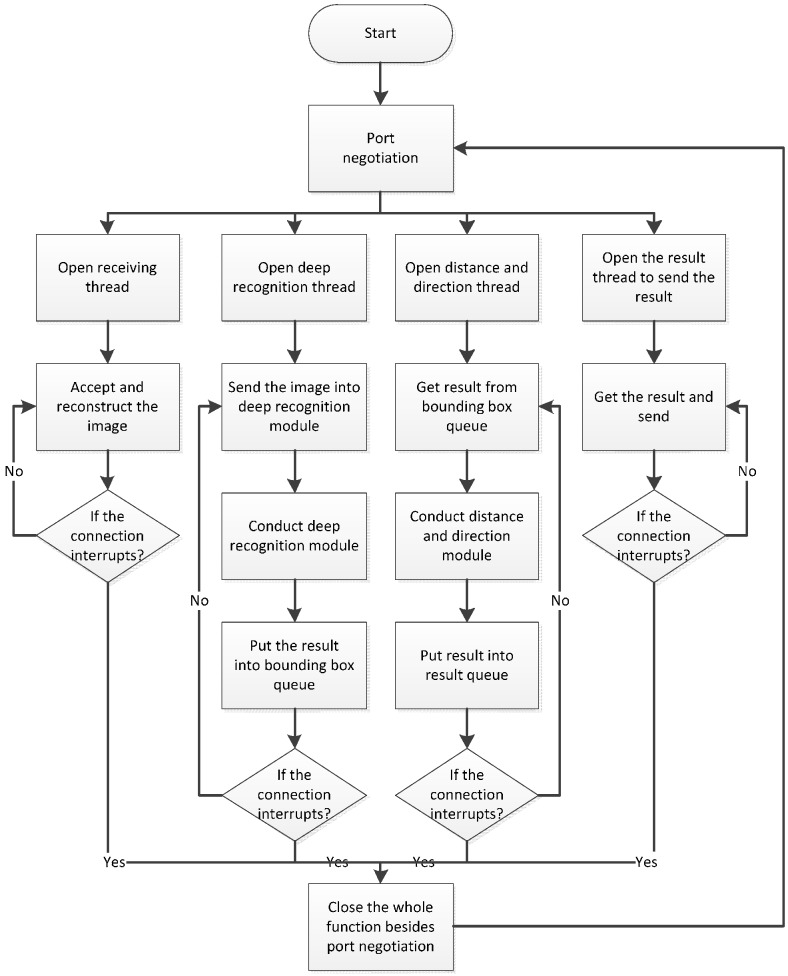
Software process of the server.

Figure 8 shows the operating process of software on the server side. After the program has been launched, the server continues to supervise the connection request from the remote smartphone. After the connection request is received, the smartphone performs port negotiation with the server to determine the working connection port number. After the negotiation is completed, the server launches four threads that receive images transmitted from the smartphone, run the deep recognition module, run the direction and distance modules, and transmit the recognition results. When the connection is interrupted, the server closes these four threads, maintaining only the port negotiation thread to wait for a connection to be made by the next user.

### 2.3. System Software Design

This section introduces the aforementioned software modules. The software design of our proposed system involves the feature recognition, deep recognition, and direction and distance modules. The feature recognition module was developed using Java and OpenCV 3.0.0, and installed in a smartphone in this study. This module provides face and stair recognition. The other two modules are executed by the server operated by an Ubuntu 14.04 Linux operating system. The deep recognition module was developed using C and the Caffe deep learning framework and accelerated using CUDA 7.5 (NVIDIA Corp., Santa Clara, CA, USA). The direction and distance module was written in Python language. The three recognition modules are described in the following subsections.

#### 2.3.1. Feature Recognition Module

After a series of interviews with visually impaired people, our system was determined to detect faces and stairs using a simple feature recognition algorithm. The feature recognition module can honor basic requests made by visually impaired users and runs in real time on a smartphone without an Internet connection. When a user is in an area with poor signal coverage or his or her smartphone loses its network connection, the module operates normally to provide recognition results. Because the computing capability and system memory of a smartphone are far slower and smaller than those of a computer, a smartphone cannot efficiently run the deep learning algorithm. Thus, the deep recognition module is shifted to the server side.

In addition to the deep learning algorithm, there are three other methods for stair recognition. In the first method, the system captures color and depth information by employing the depth camera, and it uses the results to determine whether an obstacle is a flight of stairs. Although the accuracy of this method is high, more information is required than for the other methods. In the second method, the system judges an image based on manual features defined by the user. This method does not require a large amount of information, but it is less accurate than the first method, and the false positive rate of recognition is high. In the third method [23], the system performs stair recognition on a usual color image through the feature classifier, which is trained by the machine learning algorithm. The smartphones used in this study could not capture depth information; therefore, the first method was unavailable. Moreover, the third method has a higher recognition rate and lower false positive rate of recognition. Therefore, the third method was chosen as the final method for stair recognition.

Our system implements offline image recognition through the Haar feature and histogram of oriented gradients (HOG) feature to sort through detected objects. The Haar feature-based image recognition algorithm [23] is used to detect stairs in front of the user, whereas the HOG feature is adopted to detect human faces in front of the user. 

The feature recognition module is used for the following three main reasons: First, our system can provide basic services in mobile environments, and the inclusion of the feature recognition algorithm enables the system to maintain its functionality with no Internet connection. Second, the feature recognition algorithm provides extensibility. Although the system currently offers only face and stair recognition on a smartphone, it can detect other types of obstacles by training the classifier with more labeled images. Finally, compared with the deep recognition algorithm, the feature recognition algorithm is faster and more efficient, and therefore, it is easier to implement and use on a smartphone with low computing capability. 

**Figure 9:**
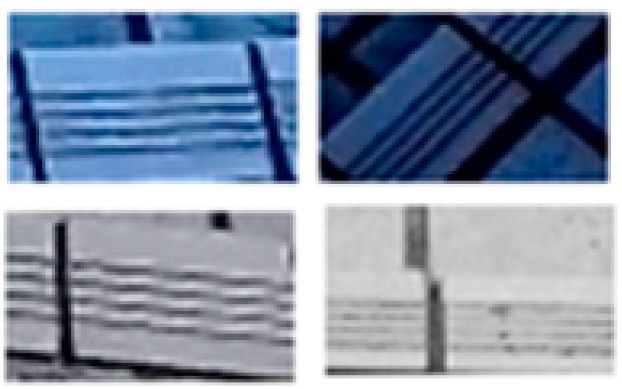
Positive training samples.

The classifier of the feature recognition module is generally required to recognize a target object. Thus, target object images should be collected in advance, after which an appropriate training algorithm is used to train the classifier. In this study, the Adaptive Boosting (AdaBoost) algorithm was mainly used to train the Haar feature classifier, and several weak classifiers were cascaded to form a cascade classifier. The cascade classifier refuses objects that weak classifiers classify as unwanted targets. This design can prevent additional computing waste. Because there are many types of stairs, using a single algorithm to recognize all stairs is not easy. Therefore, in our system, stairs are defined as stairs with antislip strips. We collected 1935 positive samples and 2630 negative samples in 48 × 28 pixels images. The samples were processed using the discrete AdaBoost algorithm to train the Haar feature classifier. Several positive samples are shown in Figure 9.

**Figure 10:**
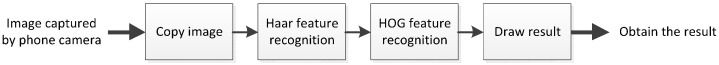
Feature recognition module operating procedure.

After a smartphone has captured a frontal image, the image is sent to the feature recognition module. First, the smartphone reproduces the image. If the smartphone is in online mode, the original image is sent to the remote server. If the smartphone is in offline mode, the original image is not sent anywhere. Subsequently, the module processes the reproduced image to detect stairs through the Haar feature classifier and recognize human faces through the HOG-trained classifier. Eventually, the smartphone constructs a recognition result on the clone image. The procedure is shown in Figure 10.

#### 2.3.2. Deep Recognition Module

There are two typical methods for implementing image recognition. The first method performs recognition according to feature points. This method processes different feature descriptions and then captures feature points based on these descriptions. Finally, a feature point matching algorithm is utilized to perform feature matching. The second method uses machine learning algorithms to train a model that has recognition capability. This method first detects fulfilled features and then uses machine learning to train a recognition model.

Traditional manual feature recognition algorithms have two drawbacks. First, preprocessing requires a large amount of time to extract features and search for matches. When object categories grow, this type of algorithm becomes inefficient. Furthermore, manual feature matching may extract erroneous features, engendering a low recognition rate. Consequently, investing large amounts of time in surveying every object's feature points is essential. Previously, the feature recognition algorithm could solve only one specific object’s recognition problem. By contrast, our system must recognize multiple object types to satisfy our specifications. Each object type requires an individual algorithm to achieve object recognition, and this considerably increases the complexity of maintenance. For the aforementioned reasons, our proposed system requires a systematic method to solve this problem.

To obtain multiple object recognition systematically, a deep learning method is implemented to automatically learn and extract feature values, drastically shortening the extraction time of object features. For image recognition, Convolutional Neural Networks (CNNs) are the most effective among all current deep learning architectures. CNNs have the advantages of decreasing image noise, increasing signal features, and simplifying neural networks. Thus, CNNs are being applied extensively in deep learning, and they represent the most popular model architecture applied by most people. Although our system conducts estimations to solve object location and classification problems, the usual CNN algorithm only solves object classification problems. The typical object location method is to apply a sliding window and image pyramid while searching through an image, thereby determining the location of individual objects. However, the recognition speed of this method is extremely low.

Girshick et al. and Ren et al. have proposed many advanced deep learning models [24,25,26]. These studies improved deep learning models with the AlexNet architecture [27] with the objective of accelerating recognition performance and increasing the detection rate. Subsequently, the recognition problem shifted from being a regression problem to a classification problem. These studies divided the problem using the selective search algorithm [28]. Subsequently, spatial pyramid pooling [25] was designed to increase the recognition rate, thereby reducing the loss of image information caused by transformation or slicing.

After evaluating many deep learning algorithms for our system, the Faster R-CNN [29] and YOLO [30] algorithms were selected to perform systematic object recognition. These two algorithms have inherent benefits and drawbacks. The Faster R-CNN algorithm contains two networks. The function of the first network is to ensure the rough position of an obstacle, whereas that of the second network is to determine the object type. With this design, the Faster R-CNN algorithm is more accurate, but slower. By contrast, the YOLO algorithm contains only one network used to obtain an obstacle’s position and its corresponding class type; thus, this algorithm is fast. However, because an obstacle’s position is learned through regression, the accuracy of this algorithm is relatively low. No algorithm or structure achieves fast recognition and high accuracy; therefore, one of the two aforementioned algorithms is selected according to a user’s current circumstances. The Faster R-CNN algorithm is applied in the stable mode to obtain higher accuracy, whereas the YOLO algorithm is performed in the fast mode to obtain a higher processing speed. To increase the CNN’s calculation speed, our system uses CUDA to perform large matrix computation.

**Figure 11:**
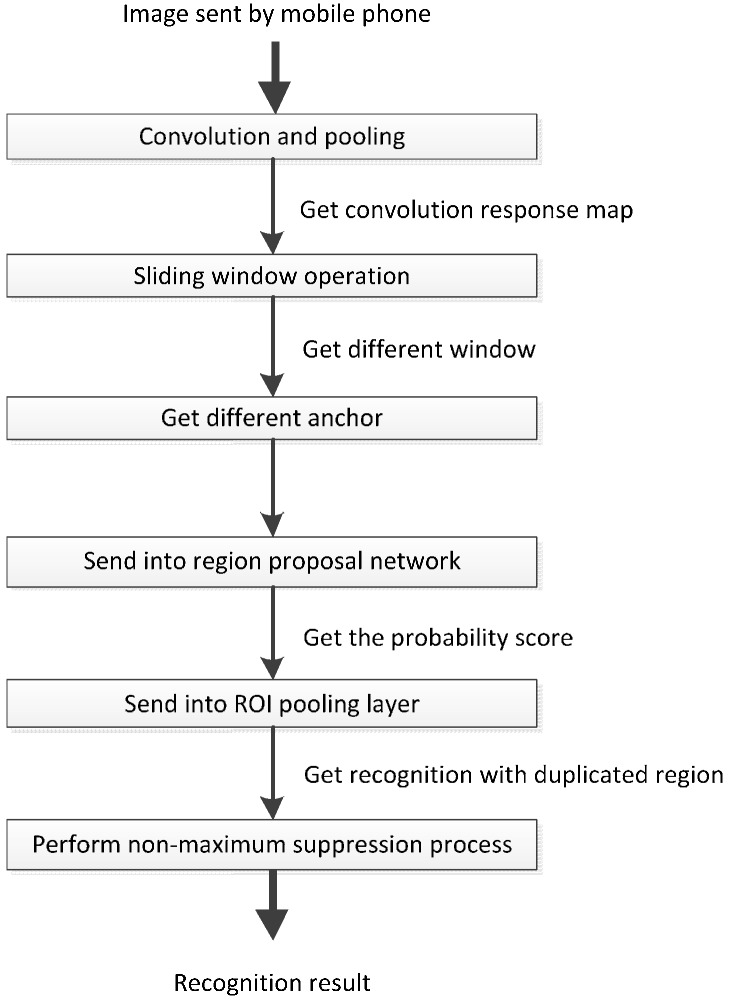
Operating process of the deep recognition module in stable mode.

In the stable mode, when the receiving thread receives an image sent from a smartphone, the image is transmitted to the deep recognition module that is based on the Faster R-CNN algorithm. After the image has been received, it is sent to the CNN model to obtain its convolutional response map. Subsequently, the deep recognition module applies sliding window scanning to the image’s convolutional response map to determine windows and anchors of various sizes and scales. Next, the module places every anchor into an area classify layer to obtain their values, thereby determining whether the candidate area is the target object. A coordinate-responded convolutional response graph is sent to a region of interest pooling layer. Finally, the non-maximum suppression algorithm is run to determine the recognized object type and its coordinates. This process is shown in Figure 11.

In the fast mode, after the receiving thread receives an image sent from a smartphone, the server sends the image to the deep recognition module that is based on the YOLO algorithm. After receiving the image, the module sends it to the CNN algorithm to directly obtain the image’s convolutional response map. There are many types of obstacles. Therefore, after the interviews with the visually impaired people, we derived the following seven obstacle categories for recognition on the server: person, car, bus, motorcycle, bicycle, potted plant, and pier.

#### 2.3.3. Direction and Distance Module

After an image has been processed by the deep recognition module, the server can obtain an object’s class ID, starting coordinates, height, and width. Recognition results are continuously sent to the direction and distance module. Finally, this module outputs the object’s category, direction, and approximate distance from the user.

**Figure 12:**
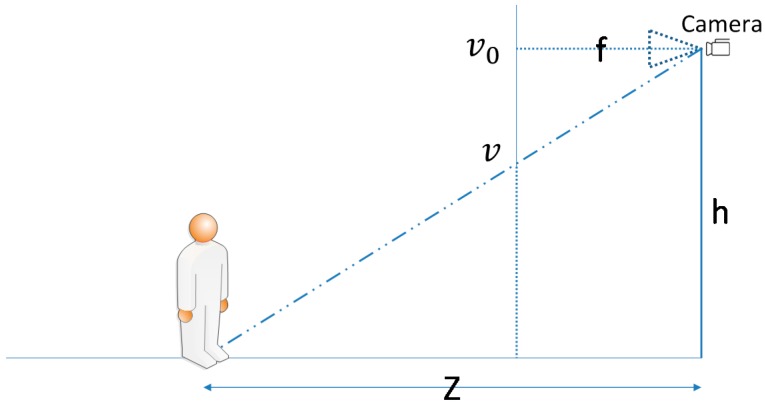
Diagram of distance calculation.

We referred to the method of Davison et al. [31] to calculate an object’s approximate distance from a user. The concept is illustrated in Figure 12, where f. is the camera focus, $k_{v}$ is the pixel density (pixel/meter), h is the camera height from the ground, $v_{0}$ is the center coordinate of the formed image, v is the target object connected to the ground coordinate, and Z is the distance between the target object and camera. Equation (1) is the formula for distance calculation:

(1) $$Z=\frac{f\;*k_{v}\;*h}{v-v_{0}}$$

This formula considers that the image of an object at an infinite distance from a user converges to a central point while the image is captured by a camera positioned at a 90° angle to the ground. Thus, every object that touches the ground must be below the center of the image to enable a triangle similar to the one in Figure 12 to be calculated. By detecting an object connected to the ground coordinate, our system can obtain distance Z without considering the object's actual size. After the direction and distance module calculates the distance, it outputs the corresponding range category according to the Z value. The sorting rule is described as follows: (a) If the calculated distance is shorter than 5 m, the range is defined as “close”; (b) if the calculated distance is between 5 m and 10 m, the range is defined as “medium”; (c) if the calculated distance is longer than 10 m, the range is defined as “far.”

**Figure 13:**
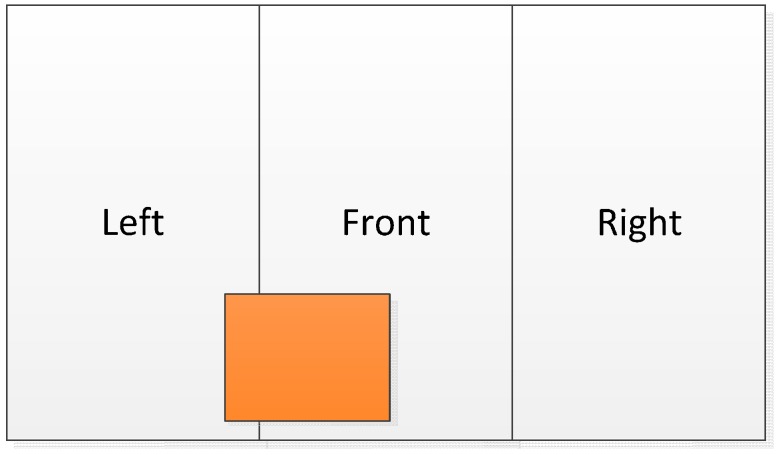
Directional judgment segments.

The module’s directional recognition function splits the image into three equally sized left, middle, and right segments. This module detects the specific segment where the object’s center coordinate is located and then outputs the corresponding segment. Figure 13 shows a direction schematic, where the central point of the orange object is in the middle segment of the image. Under such circumstances, our system notifies the user of an obstacle in front of him or her.

## 3. Experiment Design

For on-site testing, four visually impaired students (three women and one man; age: 19–22 years old) were invited to test our system. The detail characteristics of each participant are shown in the Table 1. Each participant has different major demand in the experiment. For the degree of visual impaired, all participants have partial level. Moreover, all participants didn’t previously have any experience of using other similar guiding system.

About the experiment process, the participants were required to turn the camera lens of smartphone to the front side and walk through campus from a specified building to the bus stop near the school gate. Each experiment was arranged between noon and dusk, and it lasted about several minutes. Before experiment, a tutorial would be provided to let the participant know the usage of the system. Except to the Participant 4, we accompanied all participants walking to guarantee the participant's safety during using the system. 

## 4. Results and Discussions

This section describes and discusses the smartphone program, recognition results, recognition rate, and test results.

### 4.1. Smartphone Program

The mobile application (APP) in this study was mostly developed using Java language on the Android platform. The APP consists of recognition and setting parts. The recognition part contains the feature recognition module to perform face and stair recognition, whereas the setting part provides a user interface to assist visually impaired users to quickly and conveniently control the APP settings.

When a user uses the APP for the first time, the smartphone voices the basic operating steps to enable the user to quickly understand how to operate the APP. After the APP is launched, the main menu containing the start-up button and settings button is the first page shown to the user. When the user clicks on the blue settings button on the lower right side of the screen, the settings interface is displayed. Because a first-time user is not familiar with the operation or settings of the APP, he or she should ask for help from friends or guides in changing the settings to those that meet his or her individual requirements. The text size of the program is larger than that of the original smartphone APP. Through this design, a visually impaired user can lightly revise the settings by using an auxiliary magnifier. A voice reminder is provided to notify the user about which page is currently being displayed. Because the settings function is used less frequently compared with the direct use of the recognition function, most of the screen area is reserved for the start-up button, which is bigger than the settings button to enable the user to more easily initiate the recognition procedure. After the start-up button is pressed, the recognition functionality will start working. 

After entering the settings page, a user can control the settings by swiping particular items with his or her fingers. The first page contains options for users to choose whether they want to listen to direction- or distance-related information. The second page, namely the transmission mode page offers the option of stable mode or fast mode. If the stable mode is selected, images are transmitted to the server through the TCP; otherwise, images are transmitted through the RTP.

**Figure 14:**
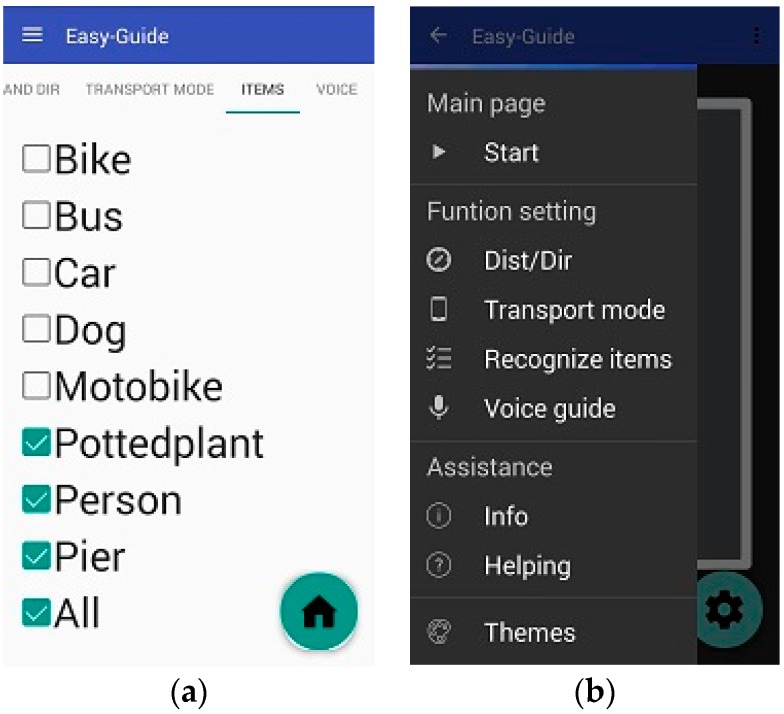
Snapshots of Android program: (**a**) settings page for obstacle type selection; (**b**) settings options with black background and white words.

On the third settings page, users can select which obstacle types they want to be alerted of Figure 14a. The final settings page contains language settings, enabling users to select the language of the voice instructions. Currently, the system provides only Chinese and English. In addition, the vocal speed can be selected and the voice can be switched on and off on this page.

On the main page, users can touch the upper left part of the screen to display the settings in the side column, which can also be displayed by swiping right on the screen. In addition, users can choose between the aforementioned four options and adjust the aforementioned items. To satisfy various visual habits, the system offers two interfaces, involving a black background with white words (Figure 14b) and a white background with black words.

### 4.2. Recognition Result and Recognition Rate

To meet various user needs to a sufficient extent, according to the answers obtained from the interviews with visually impaired people, this system is designed to recognize seven common obstacle types. In this study, the system was trained using an Internet model that had been trained by an image dataset called ImageNet [32]. We intend to train the system again using images in Pascal [33]; however, Pascal has the following three drawbacks. First, piers are not present among the 20 image categories in Pascal. Second, although images in Pascal were collected from various locations worldwide, none have been captured from experimental environments such as the ones used in this study. Third, the images in Pascal were captured using high-resolution cameras, whereas the images recognized by the system in this study are captured using smartphones. The difference in scale between images captured using a high-resolution camera and those captured using a smartphone would influence the recognition results. To overcome these drawbacks, images captured by ourselves were used in this study to train the classifier, thereby enabling the training model to be closer to a real situation. Therefore, 1710 images were taken in and around the campus, and each obstacle in these images was labeled with its location and type. 

**Figure 15:**
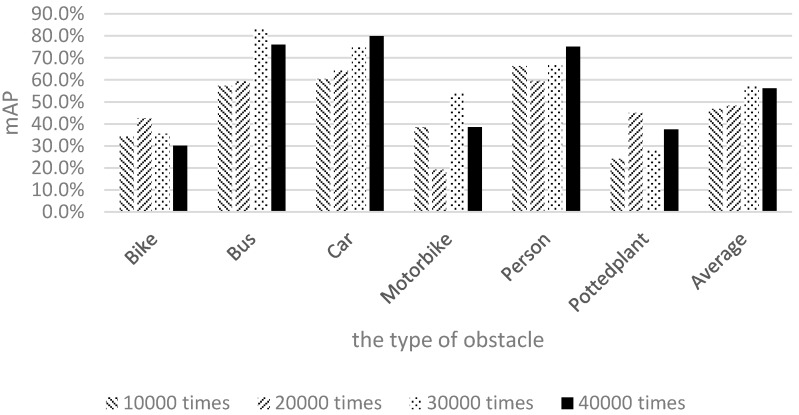
Results of using only Pascal images for training.

This study adopted mean average precision (mAP) to evaluate the effectiveness of depth learning. Additional 800 images captured in and around campus were used for testing. We conducted the training process 10,000, 20,000, 30,000, and 40,000 times to evaluate the differences in the mAP values. Figure 15 shows the results of using only Pascal images for training.

**Figure 16:**
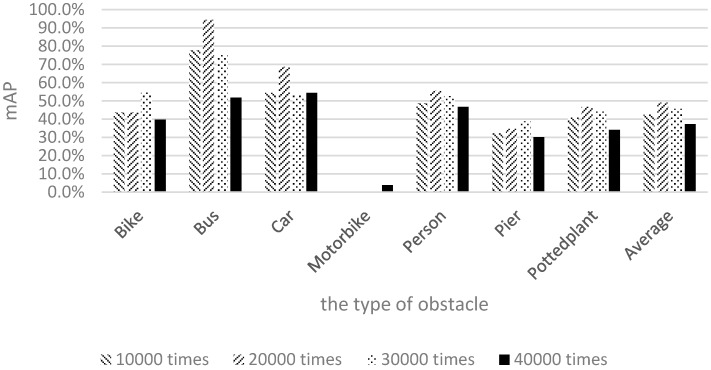
Results of using only 1710 images captured by the researchers of this study.

The shapes and appearances of labeled categories in the Pascal dataset were observed to differ from those of the categories on our campus. Therefore, among the six categories, the mAP values of bicycles, motorcycles, and potted plants were relatively low. To allow the image data to correlate more closely with the actual appearances of objects on our campus and gain more training data for piers not present in the Pascal dataset, 1710 local images captured by the researchers of this study were used for training. The results are shown in Figure 16.

**Figure 17:**
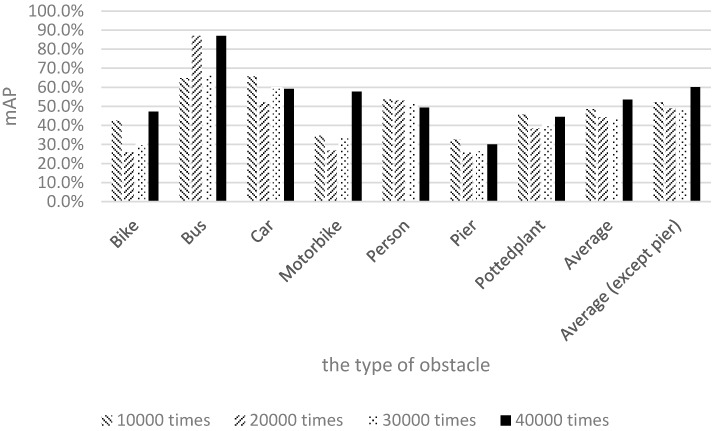
Results of using Pascal images and 1710 images captured by the researchers of this study.

Figure 16 shows the mAP of motorcycles to be very low; this is because few people ride motorcycles to school. Therefore, the quantity of motorcycle samples was lower than those of the other categories, yielding a lower recognition rate for motorcycles compared with the other categories. To achieve our goal of raising the number of samples for retraining, Pascal images and images captured by the researchers of this study were mixed together for training and testing. Figure 17 illustrates the training and recognition results of using a mixture of both image types.

Figure 17 shows that the recognition rate for buses nearly reached 90% after 20,000 training iterations. The recognition rate for piers was more than 20%, and the recognition rate for motorcycles was close to 60% after 40,000 training iterations. The experimental results show that the average recognition rate of the seven categories reached 55% after 40,000 training iterations. 

This study discussed the factors that influence the recognition rate. In the training data of the experiments, the Pascal images and our own 1710 images were merged to compose the final input. Because the Pascal dataset contains no pier training data, the number of pier training images was considerably lower than those of the other image categories. Moreover, the pier training samples adopted in this study were captured using a smartphone, thereby yielding reduced photo quality for pier images and an inability to collect accurate and abundant information for the pier category. Consequently, the pier recognition result was lower than those of the other obstacle types. Another category with a lower recognition rate was potted plants. In the Pascal dataset, the definition of potted plant is more inclusive; an item with green leaves and a container is regarded as a potted plant. By contrast, the definition of potted plant in our experimental environment was more exclusive, with a potted plant being defined as an object with a circular brown container and a height not exceeding the height of an average person. Although these two definitions are similar, they are not the same, and the different definitions could lead to many green objects being considered potted plants. Thus, frequent detection of potted plants occurred in the testing process, yielding a relatively low recognition rate.

The previous discussion reveals that the pier and potted plant categories had low performance due to the quantity of training data and the variations in definition. After excluding piers and potted plants and conducting a second comparison, we observed that the average recognition rate could reach 60% after 40,000 training iterations, thereby achieving the goal [29].

**Figure 18:**
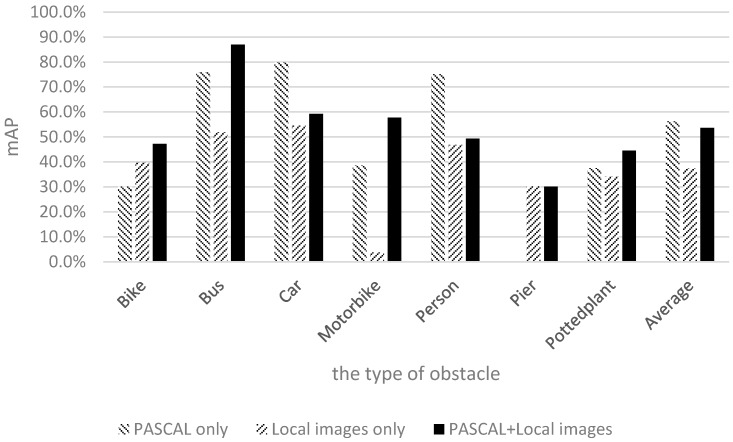
Comparison between Pascal-based, local, and mixed datasets after 40,000 training iterations.

Figure 18 shows the comparison of the recognition effects associated with the three datasets after 40,000 training iterations. As shown in Figure 18, although the mAP of the mixed images was slightly lower than that of the Pascal images, it was within a permissible range. The Pascal mAP was the highest because the pier class was excluded. However, the recognition of piers is crucial for visually impaired people. Therefore, the combination of mixed images along with 40,000 training iterations could more closely meet user needs. Retraining helps to increase the effectiveness of recognition and achieve our goal.

Because the experimental environment was organized on campus, the training process involved fewer cars and motorcycles, rendering the recognition rate not ideal. Further research could increase the number of samples in the training sets or process the samples through high-end graphic processors to improve the learning levels, thereby improving the recognition rate of each object.

**Figure 19:**
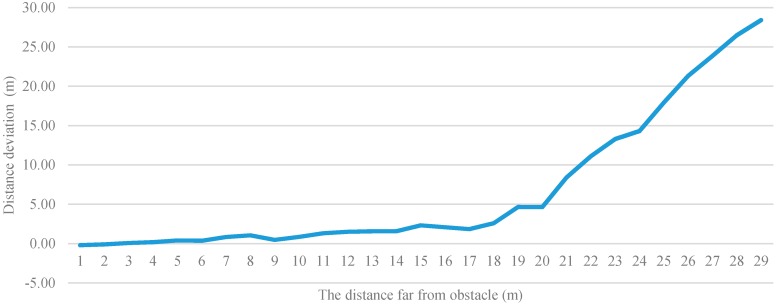
Deviation between calculated distance and actual distance.

Figure 19 shows the deviation between the calculated distance and actual distance. The vertical axis is the distance deviation and the horizontal axis is the distance from the user to the obstacle. In the experiment, deviations were recorded for each time the user walked back 1 m. Subsequently, the deviations were plotted on a line graph with meters as the unit of measurement. Figure 19 indicates a distance of 10 m to be a practical limitation in this study. If the actual distance is more than 10 m, the deviation would increase significantly, and if the actual distance is less than 10 m, the average deviation is within 10%. We divided the distance into far, medium, and near. Therefore, under a valid recognition distance, if the average deviation is within 10%, the system has already reached the effects of judging the distance.

### 4.3. Test Results

**Figure 20:**
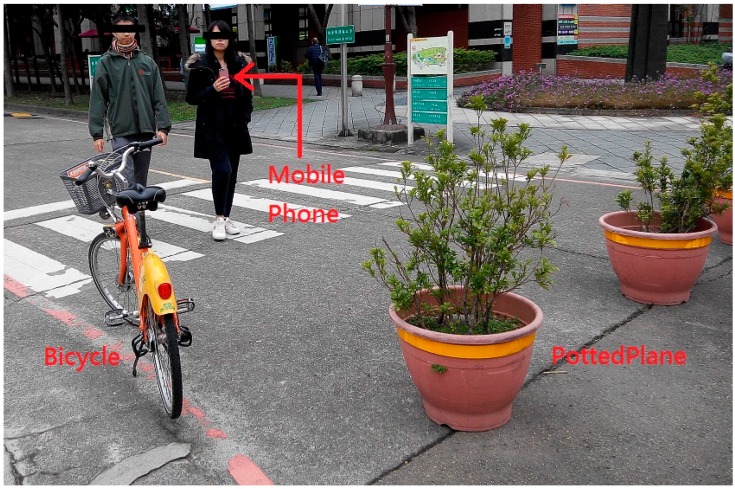
Photo of on-site testing.

Figure 20 shows on-site testing in action. After completing the test, the users deemed this system highly effective for avoiding obstacles while walking. Regarding the design of the settings page, the user interface of the smartphone was modified according to each user’s preferences. By the estimation of the battery consumption, this proposed system can provide the service in two hours with the corresponding smartphone. 

**Figure 21:**
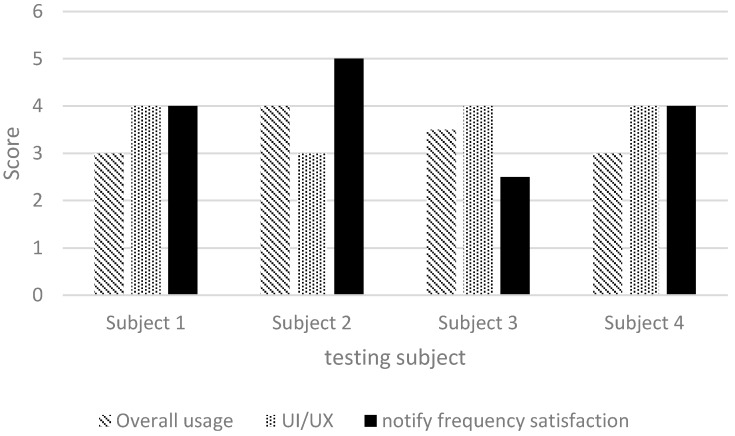
Questionnaire results.

In addition to on-site testing, we designed a simple questionnaire for users to provide feedback. Each questionnaire item was to be scored from 1 to 5 points, with 1 point representing the lowest possible score. The following items were included: (1) overall impression; (2) user interface and user experience; and (3) alert frequency. The results are shown in Figure 21, indicating that the scores were mostly higher than 3 points (Figure 21), thereby verifying the high effectiveness of our system in assisting visually impaired people. The alert frequency also met user expectations.

### 4.4. Discussion

Table 2 shows the difference between the stable mode, fast mode and offline mode. As Table 2 mentions, the stable and fast modes have higher accuracy since they both use deep learning methods to solve the obstacle recognition problem. Though the offline mode cannot provide high reliability and accuracy, its processing speed is very fast and it can also keep the reliability and accuracy at medium level. In the aspect of process speed, the stable mode costs more time due to computing amount of Faster R-CNN, the fast mode can speed up for a little by using lower complexity of YOLO. The biggest advantage of the deep learning method is that it can deal with more object types. On the other word, the offline mode cannot reach the goal. However, the offline mode can accomplish the task with the fastest speed because the smartphone does not need to transmit images to server. 

Table 3 shows the comparison between our proposed system and the other related systems. The sensing types of the systems include depth image, general image, RFID, ultrasonic sensor, and GPS. Except for the systems Kassim et al. and Bahadur et al. proposed, the other four systems can achieve high accuracy when judging distance. However, only our proposed system provides the ability to recognize several kinds of obstacles. In detail, it can recognize about seven types of obstacles. Regarding the convenience, our proposed system and [16] are more convenient than the other four systems. Comparing the sensing devices of all system in Table 3, our system has the unique advantage of only using the general smartphone as the sensing device, unlike other systems which need to use specific devices. Thus, the system we proposed has high convenience. The user doesn’t need to wear other specific devices or hold a modified cane. The user only needs to hold the smartphone and face to the front, and the smartphone can directly tell the result by voice. The drawback of our system compared to other non-image-based systems [8,16,22] is the large information computing burden. This is an inherent limitation for all image-based recognition systems. In summary, the system in this study can provide sufficient and useful information to visually impaired people.

Through the comparison in Table 3, our proposed system has the ability to judge distance, directional judgment and obstacle recognition. The high convenience and broad application environment are the best contributions compared with other similar type of devices. In the comparison summary, our proposed system has more advantages than the others.

## 5. Conclusions

This study designed a user-friendly guidance system for visually impaired people that involves a smartphone and server. When the system is in use, the smartphone continuously transmits images of the scene in front of the user to a server through 4G technology or a Wi-Fi network. Subsequently, the server performs the recognition process. The final result is transmitted back to the smartphone, which provides the user with obstacle information through voice notifications. This system can not only inform the user of the type of obstacle in front of him or her, but also reveal the approximate distance between the user and the object. Compared with the more traditional guidance method involving a modified guide cane, the proposed system provides more information for the user. After experiments were conducted, a training model with a combination of Pascal images and local dataset images provided an optimal recognition effect, enabling the system to achieve a 55% recognition rate. After excluding piers and potted plant obstacle categories, which are easily influenced by specific conditions, and conducting the computation again, the system reached a 60% recognition rate. Finally, this study issued questionnaires to visually impaired people, the results of which showed that this system provides fundamental recognition and is more effective than a guide cane. 

Compared with traditional guiding systems that are only applicable under specific circumstances, the usage of our system is unlimited. The proposed system provides more information than does a conventional guiding system, in addition to being being applicable to different environments. Thus, specific areas and special conditions are not necessary. Actual tests and experiments verified that the obstacle recognition function and user interface meet user requirements. The system can immediately warn users of obstacles ahead. Users in unfamiliar areas can instantly know the situation in front of them through the APP developed in this study. Thus, visually impaired people can quickly become acquainted with their surrounding environment, and be prepared to react to any circumstance occurring at any time. In the future, to provide information on more types of obstacles and more accurate recognition, a broader range of obstacle images and a high-end server equipped with a more powerful graphics processing unit could be used to increase the number of recognition categories and the recognition rate. On the other hand, the feature recognition has extensibility. More recognition services of other object types can be added when the computing ability of smartphone improves. These improvements could provide more information without needing wireless connections.

## Figures and Tables

**Table 1:** Personal and experimental details of each participant.

	Participant 1	Participant 2	Participant 3	Participant 4
Gender	Female	Female	Female	Male
Age	19	20	20	22
Degree of visual impairment	Partial	Partial	Partial	Slight
College of major study	Society	Law	Society	Society
Experience of using other similar guiding systems	No	No	No	No
Experiment time period	Noon	Afternoon	Afternoon	Dusk
Experiment duration (min)	7	8	8	8
Obstacle types	Person, car, bus, motorcycle, bicycle, pier	Person, bicycle, potted plant, pier	Person, car, bus, bicycle, potted plant, pier	Person, car, bus, motorcycle, bicycle, potted plant, pier
Explain usage before experiment	Yes	Yes	Yes	Yes
Accompany participant walking	Yes	Yes	Yes	No

**Table 2:** Comparison of different modes.

	Online Mode (Stable Mode)	Online Mode (Fast Mode)	Offline Mode
Accuracy	High	Medium	Medium
Reliability	High	Medium	Medium
Spend time to transmit image to server	Yes	Yes	No
Process speed	Slow	Normal	Fast
Number of recognition of obstacle types	Many	Many	Few

**Table 3:** Comparison between our proposed system and related studies.

	Hoang et al. [6]	Kassim et al. [7]	Khine et al. [8]	Bunnan et al. [16]	Bahadur et al. [22]	Our System
Input data	Depth image	General image	RFID	Ultrasonic sensor	GPS	General image
Ability to judge distance	Yes	No	Yes	Yes	No	Yes
Accuracy of distance judgement	High	None	High	High	None	High
Ability to judge obstacle types	Yes	No	No	No	No	Yes
Number of recognized obstacle types	1	0	0	0	0	7
Amount of information	Much	Much	Few	Few	Medium	Much
Sensing device	Specific device	Specific device	Specific device	Specific device	Specific device	General smartphone
Convenience	Low	Low	Low	High	Medium	High
Environments for usage	Wide	Narrow	Narrow	Wide	Narrow	Wide
